# Limited correlation between physical performance and patient‐reported outcomes at 1‐year follow‐up after anterior cruciate ligament reconstruction

**DOI:** 10.1002/jeo2.12071

**Published:** 2024-07-17

**Authors:** Torsten Grønbech Nielsen, Ulrik Dalgas, Martin Lind

**Affiliations:** ^1^ Department of Orthopaedic Surgery Aarhus University Hospital Aarhus Denmark; ^2^ Department of Physiotherapy and Occupational Therapy Aarhus University Hospital Aarhus Denmark; ^3^ Exercise Biology, Department of Public Health Aarhus University Aarhus Denmark

**Keywords:** anterior cruciate ligament, hop test, patient‐reported outcome, return‐to‐sport, strength test

## Abstract

**Purpose:**

The majority of anterior cruciate ligament reconstruction (ACLr) patients wish to return to sport. Clinical evaluations after ACLr often do not include physical testing, making it difficult to determine the patient's readiness to return to sport. Thus, it would be helpful to identify easily assessable factors associated with physical function in ACLr patients that could inform planning of patients' return to sport. This study sought to evaluate the associations between physical test performance in ACLr patients and known ACL injury risk factors, knee laxity and patient‐reported outcomes at 1‐year follow‐up.

**Methods:**

The cohort included isolated primary ACLr patients operated between 2009 and 2014. Patients were invited to a 1‐year visit to clarify their readiness to return to sport. A test battery was performed, including clinical evaluation, patient‐reported outcomes and three physical tests, from which the Leg Symmetry Index (LSI) was calculated. Multivariate regression analyses were performed for each of the physical tests, including known risk factors, clinical outcomes and patient‐reported outcomes. Laxity <3 mm, pivot shift = 0, Knee Injury and Osteoarthritis Outcome Score (KOOS) sport >75, International Knee Documentation Committee (IKDC) >75.9, and Single Assessment Numeric Evaluation (SANE) >92.7 were applied as cut‐off values for good versus poor status.

**Results:**

A total of 480 ACLr patients were included in the study. Laxity <3 mm had a negative impact on the single‐hop LSI, whereas a pivot shift = 0 or IKDC >75.9 had a positive impact on the single‐hop LSI. Age <20, a pivot shift grade of 0 and KOOSsport >75 were positively associated with the triple‐hop LSI. Finally, age <20 and IKDC >75.9 were positively associated with the leg extension strength LSI.

**Conclusions:**

Age, sagittal laxity, pivot shift and patient‐reported outcomes were associated with physical test performance 1 year after ACLr. However, the associations were not completely uniform and strong, so information on age, sagittal laxity, pivot shift and patient‐reported outcomes cannot replace a return‐to‐sport functional test battery in determining when it is safe to return to sport after ACLr.

**Level of Evidence:**

Level III.

**Abbreviations**
ACLr: anterior cruciate ligament reconstructions
ADL: activity of daily living
CIs: confidence intervals
DKLR: Danish Knee Ligament Reconstruction Registry
IKDC: International Knee Documentation Committee
KOOS: Knee Injury and Osteoarthritis Outcome Score
LSI: Leg Symmetry Index
*n*
: numbers
PASS: Patient‐Acceptable Symptom State
PROMS: patient‐reported outcomes
SANE: Single Assessment Numeric Evaluation
SD: standard deviation

## INTRODUCTION

In Denmark, approximately 2000 anterior cruciate ligament reconstructions (ACLrs) are performed each year (34/100,000 persons/year [2000 ACLr/5.900000 inhabitants]) [17]. More than 80% of all ACLr patients are injured while participating in sport activities, and the majority of ACLr patients strongly wish to return to their sport, which is one of the indications for performing an ACLr [17].

All physicians who perform ACLr in Denmark must register and evaluate their ACLr patients both pre‐ and perioperatively and at a final 1‐year follow‐up [25]. The final follow‐up consists of a clinical evaluation, which involves the following: instrumented knee laxity assessment, Lachman test, Pivot shift test, knee flexion/extension movement and registration of postoperative complications [6, 17, 34]. However, the clinical evaluation includes no physical testing, and therefore it is difficult to determine if the patient is ready to return to sport after ACLr. Different physical test batteries and guidelines have been developed to help inform this decision [35]. These physical and functional test batteries include different hop, running and strength tests. Optimally, these tests should provide important information on deficits relevant to exercise after ACLr. However, no previous studies have evaluated the correlations between physical performance tests and relevant clinical and patient‐reported knee outcomes to identify risk factors that are correlated with return‐to‐sport criteria.

At Aarhus University Hospital (AUH), the procedure for the 1‐year follow‐up was changed from being performed by the physicians to being performed by independent sports physiotherapist. The reason for this change was that the physicians' self‐assessment of their own performance can cloud their ability to objectively evaluate and critique their own work. As part of this change, three physical tests and two patient‐reported outcomes (PROMS) were included in an extended test battery. The inclusion of the single‐legged hop for distance, the triple‐legged hop for distance [26], the leg extension strength test [3] and the PROMS International Knee Documentation Committee (IKDC) [12] and Single Assessment Numeric Evaluation (SANE) [36] scores allow physiotherapists to collect more information to better guide patients regarding their return to sport.

No existing studies have performed this type of analyses on such a large and homogeneous cohort of ACLr patients. Thus, this study will extend the current literature by elucidating relevant risk factors, helping to inform both clinicians and patients regarding the safe return to sport. Could PROMS and clinical outcomes replace functional testing in guiding safe return to sport?

The aim of this study was to evaluate the correlation between physical tests (single‐legged hop test, triple‐legged hop test and strength test) in ACLr patients and known epidemiological risk factors, as well as clinical outcomes and PROMS at 1‐year follow‐up after ACLr. It was hypothesised that physical tests would be correlated with clinical outcomes, PROMS and known risk factors (female and age <20) in persons with ACLr tested 1‐year following surgery.

## METHODS

The present study is a cohort study including data from the Danish Knee Ligament Reconstruction Registry (DKLR) and the internal ACLr database at the orthopaedic department at AUH, where all data were collected prospectively. Data were extracted from DKLR, as well as the local database to enable relevant analyses linking established/explorative factors that are known to impact the return to sport. Approval from the local ethical committee was not necessary in Denmark, as the study is based on registry data (1‐10‐72‐6‐23). The study was approved by the Danish Data Protection Agency (1‐16‐02‐182‐23).

### Study population

The cohort included ACLr patients who underwent surgery from 2009 to 2014 at AUH. The primary inclusion criterion was an isolated primary ACLr, defined by reconstruction of only one ligament in the knee, the ACL, but allowing for meniscal and/or cartilage surgery. Patients who did not perform a single‐legged hop, triple‐legged hop for distance or leg extension strength test at the 1‐year follow‐up were excluded from the analyses. Likewise, patients below 18 years of age were excluded from physical testing for safety reasons.

### Data collection

After 1 year, patients are invited to a final clinical evaluation, which includes measurements of knee laxity [6, 34], knee range of motion and the registration of complications and new surgical interventions. Additionally, it is recommended that patients complete the PROMS Knee Injury and Osteoarthritis Outcome Score (KOOS) [28] at this time point to evaluate their subjective functional outcomes.

From 2010 to 2015, all ACLr patients at AUH were invited to participate in an extended test battery at their 1‐year follow‐up to investigate the 1‐year status of their rehabilitation and their current functional level. This battery included the single‐legged hop for distance [26], the triple‐legged hop for distance [26] and the power rig strength test [3]. In addition, patients were asked to complete supplementary PROMS (IKDC [12] and SANE [36]). All data from this test battery were registered in the orthopaedic department's internal database and merged with pre‐, peri‐ and postoperative data from the DKLR for each patient.

Preoperative data collected included sex (male, female) and injury mechanism (pivot sport, nonpivot sport), activity of daily living (ADL), traffic, work and others. Perioperative data collected included age at the time of ACLr, time from injury to ACLr, graft type for ACLr (hamstring, patella, quadriceps, other), meniscus and cartilage injury. Collected postoperative data consisted of sagittal laxity, pivot shift rotational instability, single‐ and triple‐legged hop for distance, leg extension power and PROMS (KOOS, IKDC and SANE).

### Outcomes

#### Single‐ and triple‐legged hop for distance

The single‐ and triple‐legged hop for distance tests were performed according to the standard guidelines [26]. Both hop tests were performed starting with the uninjured leg. Patients stood on one foot and performed a single‐ or triple‐legged hop for distance. They were instructed to hop as far as possible while landing on the same foot. Furthermore, both hands were kept behind the back, and the patients were instructed to ensure a safe landing. The test was performed three times, and the longest attempt (measured in centimetres) was recorded for each leg for both the single‐ and triple‐legged tests [26]. Two warm‐up attempts were performed for each hop test to familiarise the patients with the tests. The Leg Symmetry Index (LSI) was calculated by dividing the hop distance of the injured leg by the hop distance of the uninjured leg and multiplying by 100. A normal LSI was set to >90% [7].

#### Leg extension power test (power rig)

The Nottingham power rig was used to assess the leg extension power of each leg [3]. As with the hop tests, one leg was tested at a time, starting with the uninjured leg. The patients were seated and placed one foot on a footplate. The distance between the seat and the footplate was set according to the manufacturer's guidelines [3]. The patients were instructed to push down the footplate as fast as possible. Two warm‐up attempts were allowed to familiarise the participants with the test. A minimum of five attempts were performed for each leg, with 30‐s rest intervals. If improvement was observed in the fifth attempt, additional attempts were conducted until no further improvement was observed (until a maximum of 10 attempts). The highest power measured was recorded. The LSI was calculated in the same way as for the hop tests, and again the normal value was set at >90% [9].

#### Knee laxity

Sagittal laxity was measured using a KT‐1000 arthrometer, which assesses translatorical movement (mm) in the knee joint between the femur and tibia [6]. The patients were divided into two subgroups based on their side‐to‐side difference (STS) between the two knees. The normal STS value was set as <3 mm, while an STS ≥3 mm was considered abnormal [17]. Rotational laxity was evaluated using the pivot shift test [34] and graded from 0 to 3. The patients were divided into two groups: negative pivot shift (grade 0, no rotational instability) and positive pivot shift (grades 1–3) [14]. Good objective outcomes were an STS <3 mm and a pivot shift grade of 0.

#### PROMS

The KOOS was initially developed for patients with osteoarthritis and consists of five subscales: pain, symptoms, ADL, sport and quality of life. Only the KOOS subscales sport and quality of life have been shown to be valid for ACLr patients [11]. The KOOSsport subscale was used in the present study, which ranges from 0 (*extreme knee problems*) to 100 (*no knee problems*) [21]. The International Knee Documentation Society Score (IKDC) was also calculated, which ranges from 0 (*extreme knee problems*) to 100 (*no knee problems*) [12]. The SANE score was used to assess the patients' overall subjective evaluation of knee function. It ranged from 0 (*worse*) to 100 (*normal*) [36].

Good PROMS were determined by scores higher than the Patient‐Acceptable Symptom State (PASS) values. The PASS scores were set as follows: KOOSsport = 75.0 points [21], IKDC = 75.9 points [21] and SANE = 92.7 points [37].

### Statistical analysis

Baseline demographics are presented as medians with interquartiles as the data are not normally distributed, means ± standard deviations (SD) (continuous data) or proportions (*n*) and percentages (%) (dichotomous and categorical data). Subgroups were created for age (<20/>20 years), sex (male/female), sagittal laxity (STS < 3/≥3), pivot shift (grade = 0/>0) and subjective outcomes in relation to PASS values (KOOSsport <75.0/>75.0, IKDC <75.9/>75.9, SANE <92.7/ > 92.7).

Multiple linear regression analysis was employed, adjusting for potentially confounding factors. For all three functional tests, the adjusted model included age, sex, sagittal and rotational laxity, KOOSsport, IKDC and SANE. Both crude and adjusted estimates are presented as means with 95% confidence intervals (95% CIs). All statistical analyses were performed using Stata Software version 17 (StataCorp). A *p* < 0.05 was considered statistically significant.

## RESULTS

A total of 1049 patients underwent ACLr performed at AUH between 2009 and 2014. Of these, 161 patients were excluded because the surgery was an ACLr revision, and 181 were excluded because no physical testing was performed. In addition, 188 patients were excluded because they underwent multiligament surgery, and 39 patients were excluded because they were below the age of 18 years. Therefore, the final cohort consisted of 480 patients.

Baseline characteristics are given in Table 1.

**Table 1:** Baseline characteristics (pre‐ and presurgery).

Patients	*n* = 480
Age (years), median (25th–75th)	24.5 (20.1–31.8)
Sex, male, *n* (%)	257 (53.5)
Sex, female, *n* (%)	223 (46.5)
Injury mechanism, *n* (%)	
Pivot sport	257 (53.5)
Nonpivot sport	138 (28.8)
ADL	44 (9.2)
Traffic	12 (2.5)
Work	5 (1.0)
Other	24 (5.0)
Time from injury to ACLr (months), median (25th–75th)	8.5 (4.8–16.3)
Graft, *n* (%)	
Hamstring	387 (80.6)
Patella	41 (8.5)
Quadriceps	12 (2.5)
Other	40 (8.4)
Meniscus injury, *n* (%)	196 (40.8)
Cartilage injury, *n* (%)	68 (14.2)

Abbreviations: ACLr, anterior cruciate ligament reconstruction; ADL, activity of daily living, *n*, numbers; SD, standard deviation.

Crude and adjusted LSI estimates for each physical test at the 1‐year follow‐up are presented in Table 2. Patients with no rotational instability or an IKDC above the PASS threshold exhibited a better single‐legged hop LSI. However, normal sagittal laxity that was negatively correlated with the single‐legged hop LSI, age, sex, KOOSsport and SANE did not have a significant impact on the single‐legged hop LSI.

**Table 2:** Crude and adjusted estimates for each physical test at the 1‐year follow‐up.

	Single‐legged hop		Triple‐legged hop		Leg extension power	
	Crude mean (95% CI)	Adjusted mean (95% CI) (*n* = 324)	Crude mean (95% CI)	Adjusted mean (95% CI) (*n* = 316)	Crude mean (95% CI)	Adjusted mean (95% CI) (*n* = 327)
LSI	91.9 (90.5–93.2)	90.0 (85.9–94.2)	93.3 (92.2–94.3)	88.4 (85.1–91.7)	91.5 (89.9–93.2)	88.6 (83.5–93.6)
Age (>20)		−3.1 (−6.5 to 0.2)		**−5.1** (**7.8 to 2.5)**		**−5.2** (**−9.6 to −0.8)**
Male sex		1.5 (−1.4 to 4.4)		2.1 (−0.3 to 4.4)		1.9 (−1.9 to 5.8)
Sagittal laxity (<3 mm)		**−4.7** (**−8.0 to −1.5)**		−2.4 (−5.1 to 0.2)		−0.6 (−4.9 to 3.8)
Pivot shift (grade = 0)		**4.8** (**1.3–8.4)**		**3.6** (**0.8–6.4)**		−3.1 (−7.8 to 1.7)
KOOSsport (>75%)		1.1 (−2.0 to 4.2)		**2.9** (**0.4–5.3)**		−1.4 (−5.4 to 2.6)
IDKC (>75.9)		**3.3** (**0.2–6.5)**		1.2 (−1.3 to 3.7)		**8.2** (**4.0–12.4)**
SANE (>92.7)		3.4 (−1.4 to 8.1)		1.0 (−2.8 to 4.9)		5.8 (−0.9 to 12.5)

*Note*: Regression model adjusted for age, sex, sagittal laxity, pivot shift, KOOSsport, IKDC and SANE. Data are presented as means (95% CI). Bold text indicates a *p* < 0.05 and a variable influence on the adjusted results.

Abbreviations: CI, confidence interval; IKDC, International Knee Documentation Committee; KOOSsport, Knee Injury and Osteoarthritis Outcome Score Sport; LSI, Leg Symmetry Index; mm, millimetres; *n*, numbers; SANE, Single Assessment Numeric Evaluation.

Significant correlations were found between the triple‐legged hop test and age, pivot shift and KOOSsport. A negative pivot shift and KOOSsport values above the PASS threshold were correlated with an improved LSI, while age >20 was correlated with a lower LSI. Sex, sagittal laxity, IKDC and SANE did not significantly influence the triple‐legged hop test.

IKDC scores above the PASS threshold were positively correlated with LSI, whereas age >20 was negatively correlated with the leg extension power LSI. Sex, sagittal laxity, pivot shift, KOOSsport, and SANE did not have any correlation with leg extension power.

## DISCUSSION

The most important finding of the present study was that age, sagittal knee laxity, pivot shift, KOOSsport and IKDC were all correlated with physical test performance 1 year after ACLr. It is well known that low age and female sex are risk factors of ACL revision [17].

The present study demonstrated that lower age is correlated with a higher LSI in three physical performance tests (Table 2). This finding could indicate that even though younger ACLr patients score higher in physical tests, other factors are important for a safe return to sport. Cristiani et al. [5] found the same association between ACLr patients under 30 compared to over 30 years old. Sugimoto et al. [31] in their recent study found no correlation between three different age groups and their physical performance test. This difference could be due to a different age categorisation than in this present study, only 6 months of follow‐up or small group sizes.

Sex was not correlated with performance on the physical tests, which is also the conclusion in the study by Reinke et al. [27] and Sugimoto et al. [31].

Sagittal laxity ≥3 mm was correlated with a higher single‐legged hop test LSI. This finding is interesting, as it would be expected that high sagittal knee laxity would be correlated with reduced knee function. The reason for this could be that patients with higher laxity and thus higher levels of subjective instability will perform more intensive rehabilitation due to the subjective feeling of knee laxity. However, neither Cinar‐Medeni et al. [4] nor Sekiya et al. [30] found any correlation between the single‐legged hop test and sagittal knee laxity.

Correlations between the presence of pivot shift and the single‐ and triple‐legged hop tests were found, as a rotationally stable pivot shift was correlated with a higher LSI. These correlations were expected, as stability is necessary in take‐off and landing. No correlation was found between pivot shift and knee strength, which was also expected, as it is a close‐chain performance test that does not require rotationally stability. A long‐time follow‐up study by Sundemo et al. [32] found no association between pivot shift and single‐legged hop for distance. The reason for this difference may be that the pivot shift and jump tests were performed 14 years apart. Their data were not used to guide return to sport, but to assess long‐term outcomes.

Cristiani et al. [5] found a correlation between KOOSsport and the single‐legged hop and muscle power tests, as patients with an LSI > 90 had a higher KOOSsport score. In contrast, no such correlation was found in the present study.

An IKDC PASS value of >75.9 points was identified as an acceptable outcome, but this could be misleading in terms of return to sport capability. Moreover, it is not clear if a PASS value of >75.9 means the patient is ready to return to sport. Logerstedt et al. found that IKDC might be a clinically relevant tool to inform the return‐to‐sport decision, but they used the higher normal values for age and sex as cut‐off values for the safe return to sport (normal range, 83–90 points). In their study, it was concluded that an IKDC score below the cut‐off was reasonably indicative of failure on the test battery (including the hop and strength tests), whereas an IKDC score above the cut‐off was not predictive of passing scores on the return‐to‐sport test battery [18].

The PASS score for SANE was set to 92.7 following Zhang et al. [37], who reported that SANE might serve as a predictor of return to sport. No correlation was seen between SANE and the physical tests in this study.

In the present study, the single‐legged hop, triple‐legged hop and leg extension strength tests were used to evaluate whether it was safe for ACLr patients to return to sport. As mentioned above, these tests are part of larger test batteries and are useful in guiding a safe return to sport [35].

The two‐hop tests are easy to use and are part of almost all test batteries guiding patients' return to sport [7], and they have been found to be suitable for ACLr patients. To the best of our knowledge, leg extension strength testing in ACLr patients using the Nottingham power rig has not been presented in other studies. Nevertheless, the power rig has been found to be reliable for measuring muscle power, with a low habituation effect [10]. An alternative to the power rig is isokinetic dynamometry [20, 24], which is commonly used evaluating the functional outcome of ACLr. Isokinetic testing is more suitable for evaluation of isometric and eccentric muscle strength testing.

Jumping and muscle power are essential when participating in sport and physical activity. Therefore, the three tests performed in the present study are relevant when guiding patients in a safe return to sport. According to Noyes et al. [23], both the single‐ and triple‐legged hop tests have low sensitivity (52% and 49%) but high specificity (97% and 94%). A low hop‐test LSI indicates that the patient is not ready to return to sport, whereas a high LSI is not sufficient to recommend a safe return to sport [22]. Therefore, hop tests must be part of a larger test battery that includes other tests, as recommended by Kotsifaki et al. [15], West et al. [35] and Barfod et al. [2].

The PASS values defined by Muller et al. were applied in this study. However, it is important to consider whether these values are relevant for guiding a safe return to sport or if they are primarily relevant to subjective knee symptom levels, that is, ‘acceptable’ patient symptoms. In a systematic review, Macri et al. [19] presented other PASS values for KOOSsport and IKDC, which were all below the follow‐up scores found in this study. Defining a specific threshold would be useful in guiding patients wishing to return to sport. PROM score reference values for a normal sex‐ and age‐matched cohort would be helpful in determining an appropriate PASS threshold for safe return to sport.

Regarding PASS values, no consensus exists regarding which LSI value best indicates a readiness to return to sport. In this study, an LSI >90% was used as the threshold for each physical test, which is standard in most test batteries [7, 29]. Interestingly, early studies found that LSI >85% was a satisfactory threshold for single‐legged hop tests for ACLr patients [1, 23]. A study from 2022 reported an LSI of 96% for single‐ and triple‐legged hop tests in a healthy cohort [29]. Another study by Kim et al. based on 1‐year follow‐up data after ACLr reported overall LSI quadriceps strength and hamstring strength values of 77% and 86%, respectively, and an LSI of 79% for the single‐legged hop [13]. These results are lower compared to the overall crude LSI values for the single‐legged, triple‐legged and leg extension power tests in this study, which were all >90%. Thomeé et al. recommended using LSI >100% as the threshold for muscle strength and LSI >90% for the hop test [33]. However, the data used by Thomeé et al. [33] were based on a comparison of contralateral uninjured legs, which is controversial. Petschnig et al. [24] compared single‐ and triple‐legged hop for distance and leg extension power results between ACLr patients after 1 year and a healthy cohort. It was concluded that the uninjured leg was within the normal range and could be used as a reference. Recently, Gokeler et al. [8], and Kotsifaki et al. [15] reported that the LSI might underestimate performance deficits and therefore should be used with caution when guiding a safe return to sport. In addition, Leister et al. measured the single‐legged hop before and after exercise to evaluate its impact on the LSI. The LSI increased from 88.7 to 91.0% for ACLr patients, indicating that exhaustion did not impact the LSI single‐legged hop test results [16].

A strength of this study is that all patients were tested by two independent sports physiotherapists and not by the physician performing the ACLr at the 1‐year follow‐up. By using an independent sports physiotherapist, physicians were not required to evaluate their own work (ACLr), which could bias the final results. Another strength is that all data were collected prospectively and covers a large, homogenous cohort of primary isolated ACLr patients. Although this is a strength, it could also be a limitation in relation to external validity of the study findings, as these might not represent a wider population of knee ligament injuries.

There are also limitations of the study. Major limitations of the study were the lack of data on which patients returned to sport, which sports they returned to and when they returned to sport. Ideally, this information should have been collected during a long follow‐up period. Finally, no information was collected on post‐operative rehabilitation patients after ACLr.

## CONCLUSION

Age, sagittal laxity, pivot shift and PROMS were correlated with physical test performance 1 year following ACLr. However, the correlations were not completely uniform and strong, so information on age, sagittal laxity, pivot shift and PROMS cannot replace a return‐to‐sport functional test battery for determining when it is safe to return to sport after ACLr.

## AUTHOR CONTRIBUTIONS

All authors contributed to the study's conception and design. Data analysis was performed by Torsten Grønbech Nielsen. Analysis and interpretation of the results were done by all authors. Ulrik Dalgas and Martin Lind were major contributors in writing the manuscript. All authors read and approved the final manuscript.

## CONFLICT OF INTEREST STATEMENT

The authors declare no conflict of interest.

## ETHICS STATEMENT

This is a registry study. The Local Ethics Committee has confirmed that no ethical approval is required (Ethical Committee 1‐10‐72‐6‐23). Data were collected from a national registry and thus an internal database informed consent was not required.

## Data Availability

Data are available on request at torsne@rm.dk.
